# Assessment of Four Portuguese Wheat Landrace Diversity to Cope With Global Warming

**DOI:** 10.3389/fpls.2020.594977

**Published:** 2020-12-09

**Authors:** Diana Tomás, Luís Pinto Coelho, José Carlos Rodrigues, Wanda Viegas, Manuela Silva

**Affiliations:** ^1^Linking Landscape, Environment, Agriculture and Food, Instituto Superior de Agronomia, Universidade de Lisboa, Lisbon, Portugal; ^2^Centro de Estudos Florestais, Instituto Superior de Agronomia, Universidade de Lisboa, Lisbon, Portugal

**Keywords:** bread wheat, landraces, heatwave, yield, grain composition, protein content

## Abstract

Wheat is a dietary staple consumed worldwide strongly responsible for proteins and carbohydrate population intake. However, wheat production and quality will scarcely fulfill forward demands, which are compounded by high-temperature (HT) events as heatwaves, increasingly common in Portugal. Thus, landraces assume crucial importance as potential reservoirs of useful traits for wheat breeding and may be pre-adapted to extreme environmental conditions. This work evaluates four Portuguese landrace yield and grain composition through attenuated total reflection Fourier transform infrared (ATR-FTIR) spectroscopy, particularly protein content, and their responses to HT treatment mimicking a heatwave. Landraces showed distinct yield traits, especially plant height and first spike grain number, and a similar pattern in FTIR spectra, although revealing differences in grain components’ proportions. Comparison between spectra band intensity indicates that Ardito has the highest protein-related peaks, contrary to Magueija, which appears to be the landrace with higher lipid content. In plants submitted to 1 week of HT treatment 10 days after anthesis, the first spike grain size and weight were markedly reduced in all landraces. Additionally, it was observed that a general increase in grain protein content in the four landraces, being the increment observed in Ardito and Grécia, is statistically significant. The comparative assessment of control and HT average FTIR spectra denoted also the occurrence of alterations in grain polysaccharide composition. An integrated assessment of the evaluations performed revealed that Ardito and Magueija landraces presented diverse yield-related characteristics and distinct responses to cope with HT. In fact, the former landrace revealed considerable grain yield diminution along with an increase in grain protein proportion after HT, while the latter showed a significant increase in spikes and grain number, with grain quality detriment. These results reinforce the relevance of scrutinizing old genotype diversity seeking for useful characteristics, particularly considering HT impact on grain production and quality.

## Introduction

Wheat (*Triticum aestivum* L.) is a major cereal consumed worldwide on a daily basis (FAO, 2017). However, the global mean growth rate of wheat is not sufficient to cover the production predicted to be necessary in 2050 (Ray et al., 2013), and one of this limitation causes is the progressive global warming (Gaupp et al., 2019). In fact, the increase in mean temperature during wheat development was predicted to reduce grain production (Asseng et al., 2014; Wang et al., 2019).

Major effects of high temperature (HT) on wheat plants include decrease in pollen viability, plant cycle shortening, as well as deterioration of chlorophyll and reduction of photochemical efficiency with consequent grain number diminution and kernel shrinkage (reviewed in Akter and Islam, 2017). Temperatures above 30°C after anthesis, in the early stages of grain filling, accelerate plant development leading to smaller and shrunken grains (Altenbach et al., 2002, 2003). This reduction in grain development time caused by heat decreases starch and protein deposition, affecting grain composition and final quality (reviewed in Farooq et al., 2011). Several reports suggested that a HT induces higher grain protein content as kernel size is smaller, and this augment seems to be more pronounced when HTs are imposed in early stages of grain filling (Corbellini et al., 1998; Daniel and Triboi, 2001; Castro et al., 2007). However, distinct stress responses were registered in different wheat genotypes commercially available with a reduction in both kernel weight and protein content in some varieties (Tomás et al., 2020a). In this context, it is particularly relevant to comparatively assess the variability of distinct commercial varieties (Pradhan et al., 2019) and also, more importantly, the old and traditional landraces, considering the eroded genetic pool of commercial varieties that resulted from decades of homogenization through breeding.

Landraces provided notable successes in crop improvement (reviewed in Dwivedi et al., 2016). Wheat landraces, defined as a traditional varieties with potential higher tolerance to biotic and abiotic stresses, present better yield stability under low input agricultural system (Zeven, 1998). Thus, landraces may constitute extremely valuable agrobiodiversity pools assuming a prominent role in the actual unpredictable weather conditions (Lopes et al., 2015; Alipour et al., 2017).

The effects of extreme heat events particularly frequent in Portugal, like heatwaves (Cardoso et al., 2019), defined as five or more consecutive days of heat in which the daily maximum temperature is at least 5°C higher than the average maximum temperature (WMO, 2015), have been studied in wheat commercial varieties. Those reports showed that HT treatments mimetizing heatwaves during grain filling leads to lower intervarietal diversity in transcription levels of genes related to grain quality and in the proportions of distinct protein fractions (Tomás et al., 2020b), as well as in grain polysaccharide composition and global protein content (Tomás et al., 2020a). Thus, it is crucial to assess the biodiversity enclosed in landraces to cope with a broad range of environmental conditions. The objective of this work was to assess the effect of a short period of HT during grain filling in distinct Portuguese landraces on grain yield and composition with special focus on protein content as one of the most determinant parameters of grain quality. Our results revealed distinct responses to HT treatment concerning most yield parameters, except grain weight, and a concordant general increase in protein content and reduction in starch amount. Additionally, landraces presenting distinct responses to HT treatment imposed during grain filling were identified.

## Materials and Methods

### Plant Material

Bread wheat (*T. aestivum* L., 2n = 6 × = 42, AABBDD) old Portuguese landraces from Vasconcellos collection, established in the 1930s of the last century (Vasconcellos, 1933), were used in this work—Ardito, Grécia, Magueija e Ruivo. These landraces were selected considering a previous study of photosynthetic rate and thousand grain weight (Scotti-Campos et al., 2011). The seeds used were obtained after 2 years of controlled propagation under equal environmental conditions of material gently supplied by EAN Germplasm Bank (Oeiras, Portugal, PRT005). Twenty seeds from each landrace were simultaneously germinated and grown in controlled conditions—8 h of dark at 20°C followed by 16 h of light period divided into 6 h increasing to 25°C, 4 h at 25°C, and 6 h decreasing to 20°C. Three weeks after germination, plants in the growth stage between 1.3 and 1.4 Zadoks code (Zadoks et al., 1974) were transferred individually to 7-L soil pots and maintained in greenhouse conditions.

When the first anther was observed in the first spike (anthesis), plants were transferred to growth chambers with the previously described conditions. A HT regime with a daily plateau of 40°C maximum temperature (Supplementary Figure 1) was imposed to subsets of 10 plants (independent biological replicates) of each landrace, 10 days after anthesis beginning–anthesis complete (Zadoks decimal code 61) (Zadoks et al., 1974) in each plant, thus occurring in distinct dates (flowering times presented in Supplementary Table 1) for each wheat landrace/plant evaluated.

After treatments, plants were kept in the greenhouse until the end of the lifecycle. All yield and grain composition analyses were performed exclusively in seeds from the first spike to guarantee identical developmental stages during HT treatments. For grain composition analyses through ATR-FTIR spectroscopy and elemental analysis, the embryo was removed from each kernel, simulating germen industrial removal procedure for flour production.

### Yield Evaluations

Yield parameters were evaluated in all plants of all varieties in both control and treatment conditions after the plants reached harvest maturity, corresponding to at least eight independent biological replicates for each genotype/condition. The parameters evaluated per plant were height, area, and number of spikes; and in the first spike were length, number of grains, and grain weight. The average weight of 10 grains (g/10 kernels) was deduced from the two later data.

Plant area was calculated through the analysis of mature plant images (Supplementary Figure 2). At the end of the growing cycle, the plant shoot system was photographed with a Nikon D90 camera using a black background for easier software segmentation, with constant light conditions and image capture parameters (exposure time, aperture, and ISO speed). Raw images were quantified using ImageJ software (United States) with Fiji platform (Schindelin et al., 2012).

### ATR-FTIR Spectroscopy

For attenuated total reflection Fourier transform infrared (ATR-FTIR) spectra acquisition, four grains of the first spike were pooled from each plant, and a minimum of eight independent biological replicates per variety and per condition (control and HT treated) were evaluated. Grains were ball-milled in a Cryomill (Retsch GmbH, Haan, Germany) after embryo removal, and all samples obtained were lyophilized overnight. Flours ATR-FTIR spectra were recorded with a Bruker-P Alpha spectrometer (Bruker, Ettlingen, Germany) equipped with a single-reflection diamond ATR (attenuated total reflection) accessory. The spectra were obtained between 4,000 and 400 cm^–1^ with a resolution of 4 cm^–1^, and each spectrum was the average of 24 scans corresponding to technical replicates. Processing of the spectra was performed with OPUS software Vsn. 8.0 (Bruker Optics, Ettlingen, Germany). The average spectra were calculated per landrace and condition and subsequently Min–Max normalized between the minimum at 1,800 cm^–1^ and the maximum between 1,800 and 895 cm^–1^. For the nitrogen (N) prediction model, the partial least square (PLS) regression model obtained previously (Tomás et al., 2020a) was used to predict the landrace samples. After prediction, 10 samples covering the obtained N range were selected for nitrogen content quantification by elemental analysis. The spectra and values obtained (Supplementary Table 2) were included in the model, and a new model (further on referred as adjusted model) was obtained and further used to predict N content, which was then used to calculate protein content using the conversion factor of 5.7x (Caporaso et al., 2018).

### Elemental Analysis

The nitrogen content was quantified in flour of 10 samples (obtained as described for ATR-FTIR analysis) at the REQUIMTE@UCIBIO-FCT-UNL analytical laboratory using a Flash EA1112 CHNS analyzer (Thermo Finnigan CE Instruments, Italy) equipped with a gas chromatography column and a thermal conductivity detector.

### Data Analysis

To compare the yield parameters and protein content between varieties, values were fitted to a linear model (*ANOVA* with one factor with fixed effects) and analyzed through multiple means comparison test (*Tukey test*). The individual effect of HT treatment in comparison with control condition for each variety was tested using *t-test*, and χ^2^ test was used to compare frequency distributions. Models were fitted in R using *aov* and *Tukey.HSD* (*agricolae* package) and *chisq.test* functions, respectively.

The principal component analysis (PCA) and clustering analysis (dendrogram) were made based on yield quantification data in RStudio using *prcomp* and *HCPC* functions, and *FactoMineR* and *factoextra* packages.

## Results

In this work, plants of landraces Ardito, Grécia, Magueija, and Ruivo were submitted to HT treatment simulating a heatwave for 1 week during grain filling stage. Yield parameters were comparatively evaluated in the end of the lifecycle in these plants and in plants kept in control conditions. The results obtained were used to compare between landraces in each condition and to evaluate the HT effects on each landrace.

### Landraces Revealed Different Responses to HT Treatment in Yield Parameters

**FIGURE 1:**
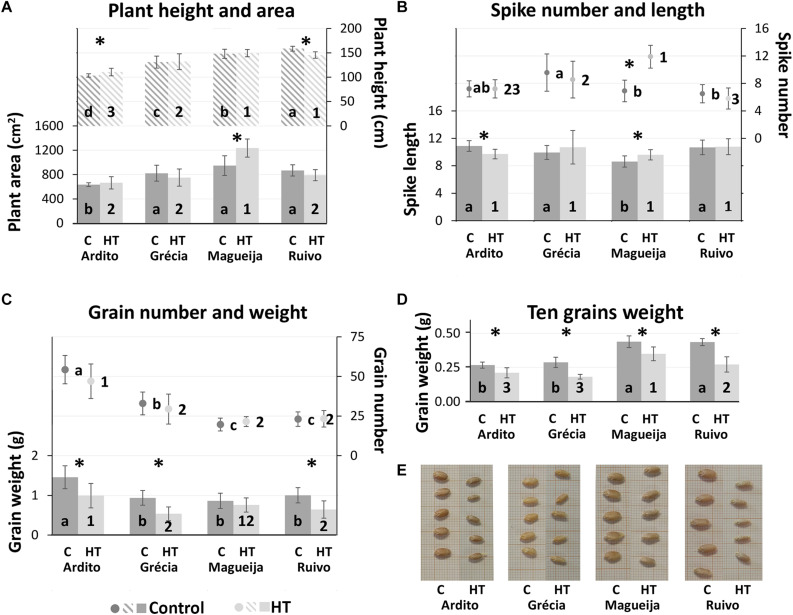
Yield parameter evaluation. **(A)** plant height (listed columns) and area (full columns). **(B)** Number of spikes per plant (dots) and first spike length (columns). **(C)** First spike number of grains (dots) and grain weight (columns). **(D)** First spike 10-grain weight. Mean values of plants kept in control conditions (dark gray) and high temperature (HT) treatment (light gray) ± standard deviation (represented as bars). Different letters and numbers indicate *ANOVA* significant differences between varieties, in control and high-temperature (HT) conditions, respectively. *t-test statistical differences between control and treatment in each variety (*p* < 0.05). **(E)** Grains from the four landraces’ plants kept in control conditions or HT treated.

The yield parameters considered are the following: (i) per plant—height, area, and spike number; (ii) in the first spike—length, grain number, grain weight, and 10 grains weight. The results obtained are summarized in Figure 1.

Plant height and area, often used as predictor of the plant biomass (Armoniené et al., 2018), calculated for control condition, revealed that Ardito was the landrace with significant lower average values in both parameters (103.42 and 549.94 cm^2^, respectively) in comparison with the other landraces (Figure 1A). However, only plant height values are significantly different among all landraces. On the other hand, HT treatment influenced significantly Ardito and Ruivo plant height, although in inverse ways. Ardito HT treated plants are 6.9% taller (110.52 cm), and Ruivo plants are 8.3% shorter (145.42 cm). Magueija HT treated plants showed an average area significantly higher (31%) than the control ones and the comparison between landraces submitted to HT conditions indicates that this value (1,166.37 cm^2^) is significantly higher than other landrace plant areas (Figure 1A).

**FIGURE 2:**
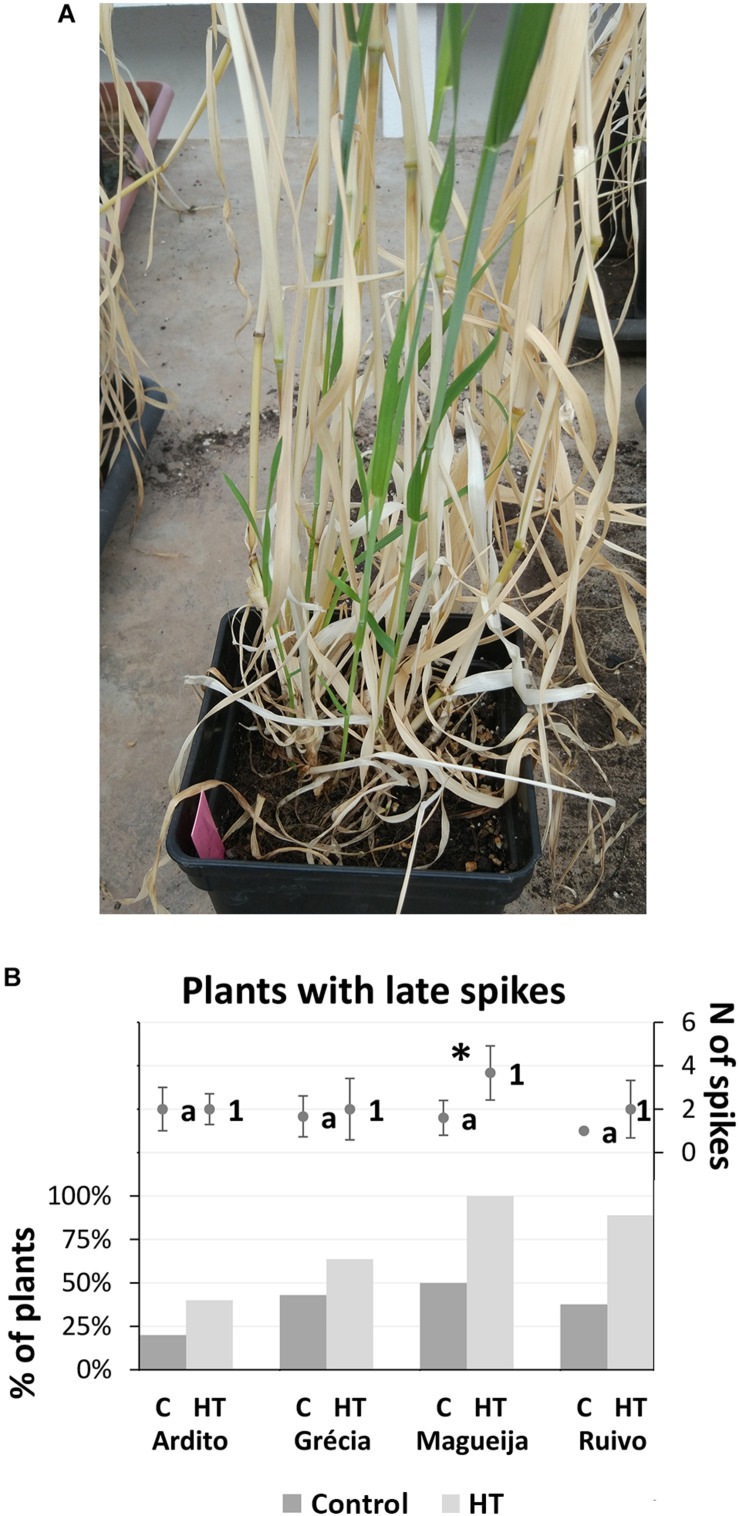
Late spike evaluation. **(A)** HT-treated plant of Magueija landrace presenting four late spikes. **(B)** Mean number of late spikes per plant in control (dark gray dots) and HT treated (light gray dots) plants ± standard deviation (represented as bars) and percentage of plants with late spikes in control (dark gray columns) and high temperature (HT) conditions (light gray columns). Different letters and numbers next to dots indicate *ANOVA* significant differences between landraces in both control and treatment conditions, respectively, and *indicates *t-test* statistical differences between control and treatment in each variety (*p* < 0.05).

Considering the average number of spikes per plant kept in control conditions, Magueija and Ruivo presented the lowest average values (6.9 and 6.5, respectively), significantly different than the highest average number (9.6) shown by Grécia (Figure 1B). The comparison of the average number of spikes between control and HT-treated plants revealed a significant difference only in Magueija, with a remarkable increase of 72%, (from 6.9 to 11.9). Also, the comparison between landraces submitted to HT treatment revealed that Magueija plants showed a significantly higher number of spikes in comparison with all other genotypes (Figure 1B). The number of spikes was moreover influenced by the appearance of new tillers after the HT treatment period, during ripening, with subsequent additional spikes (Figure 2A). These late spikes were observed in all landraces, although not in all plants, and their average number per plant as well as the percentage of plants with late spikes are presented in Figure 2B. In control conditions, the average number of late spikes per plant observed ranged between 2 in Ardito and 1 in Ruivo, but no significant differences were observed between landraces. On the other hand, Ardito was the landrace in which we detected a lower percentage of control plants with late spikes (20%), and Magueija was the landrace with higher percentage (50%) (Figure 2B). In HT-treated plants, only Magueija landrace presented a significant increase in the average number of late spikes per plant compared with the control ones, from 1.6 to 3.6. Regarding the percentage of plants with late spikes, it was observed a significant increase in all the landraces except Grécia and all HT treated Magueija plants presented late spikes.

Spike length and grain parameters (number and weight) were measured only in the first spike. Magueija plants kept in control conditions revealed to have the smallest spike with an average length of 8.6 cm, significantly lower than the other three landraces (Figure 1B), though it was the only landrace revealing a significantly larger spike in HT-treated plants in comparison with the control ones. Ardito HT-treated plants, on the other hand, showed a significant decrease in average spike length in comparison with the control plants. HT-induced alterations reduced the intervarietal variability observed regarding spike length since no significant differences were observed between landraces after HT treatment.

In accordance with the spike length, both grain number and grain weight/spike were also lower in Magueija plants maintained in control conditions (19.7 and 0.86 g, respectively) comparative to the other landraces (Figure 1C). On the other hand, Ardito was the landrace with significantly higher values in these two parameters (54.3 and 1.46 g). Although HT treatment showed no significant effect in grain number, it induced a grain weight/spike decrease in all the varieties that was statistically significant in all landraces except in Magueija. The comparison between landraces submitted to HT treatment showed that Ardito has the significantly higher number of grains/spike of all landraces (47) and a higher grain weight/spike (0.99 g) than Grécia and Ruivo. Ten grain weight (Figure 1D) allows a more accurate assessment of the distinct developmental conditions’ effects in plants’ yield. In plants kept in control conditions, Magueija and Ruivo have higher values (0.44 g) in comparison with Ardito (0.27 g) and Grécia (0.29 g). This yield parameter was significantly lower in HT-treated plants of the four landraces (between 0.17 g in Ruivo and 0.06 g in Ardito), and Magueija remains the variety with significantly higher ten grain weight in plants submitted to HT treatment. This result is clearly illustrated by the comparison of grain size presented in Figure 1E since grains from treated plants are smaller in all the landraces.

### HT Impact in Grain Composition Revealed by Attenuated Total Reflection Fourier Transform Infrared Spectra

**FIGURE 3:**
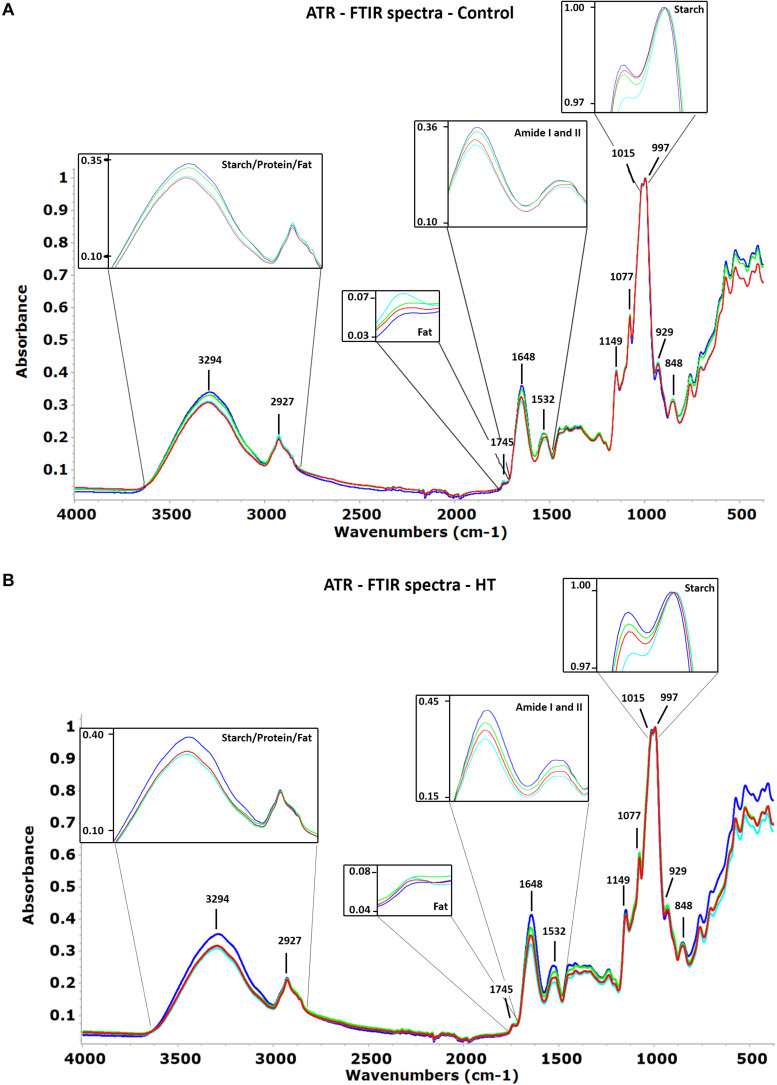
Attenuated total reflection Fourier transform infrared (ATR-FTIR) average spectra of Ardito (dark blue), Grécia (green), Magueija (light blue), and Ruivo (red) **(A)** control and **(B)** HT samples with the assignment of relevant bands. Insets magnify peaks related with starch, protein, and fat showing differences between landraces in each condition.

The spectra in the wavenumber region between 4,000 and 400 cm^–1^ obtained for the four landraces studied in each condition show no evident pattern differences, but the same bands presented intensity variations (Figure 3). The most intense bands in the region of 1,150 and 800 cm^–1^ are mainly from starch, including the most intense band of the spectra, with a maximum close to 997 cm^–1^. The band with a maximum at 2,927 cm^–1^ assigned to the stretch vibration of CH_2_ is also essentially from starch with a small contribution from proteins and lipids. The protein contribution, the second most important component of wheat grain, is clearly seen as two bands with maxima close to 1,648 and 1,532 cm^–1^ from Amide I and II, respectively. The broad band with maximum close to 3,294 cm^–1^ from O–H stretching of the starch polymer masks completely the NH band from proteins. A very weak band, in some cases only a shoulder, located at 1,745 cm^–1^, could be from C = O stretching from lipids that if present at all would be in a very small percentage.

The comparison between landraces’ min–max normalized spectra obtained for control conditions, regarding the maxima intensity at selected wavenumber bands, was performed (Figure 3A). This analysis unravels that Ardito has a more intense spectra than Magueija and Ruivo in half of the selected bands, including the band with maximum at 3,294 cm^–1^ assigned to starch polymer and Amides I and II bands. On the contrary, the average spectrum of grains from Magueija control plants was the most intense at the 1,745 cm^–1^ band (probably related with fat), and 2,927, 929, and 848 cm^–1^ starch-related bands. Globally, only for the Amide I, the four spectra are clearly separated, while for the other selected bands, at least two spectra have similar absorbance intensities.

The comparison of maximum intensity at selected wavenumber between the average spectra of grains from control and HT = treated plants, after min-max normalization is shown in Table 1. Overall, the spectra of grains from HT-treated plants are more intense than the ones obtained from grains of control plants for all four landraces. The only exceptions to these were the more intense Grécia and Magueija control spectra in the band with a maximum at 3,294 cm^–1^, mainly assigned to O–H stretching from the starch polymer. Amide I and II proteins bands are the ones that revealed more relevant differences between control and HT. In fact, the intensity of HT Ardito and Grécia spectra is much higher than the control ones in both Amide bands, as well as in Ruivo regarding Amide I band. This increase in protein content is expected to be associated with a proportional reduction in starch grain content, as these are the main components of wheat grain, and the spectra normalization was done by the more intense band at 997 cm^–1^, associated with starch. Although Ardito HT spectrum is also quite more intense than the control one in bands with a maximum at 1,149 and 1,077 cm^–1^, and Grécia presented a greater difference between the control and HT spectra in 1,077 and 929 cm^–1^ bands, in both cases, the bands are associated with starch. These alterations suggest that the proportions of distinct polysaccharides may also be altered after HT treatment. Last, based on the 1,745-cm^–1^ band, the lipid fraction increases slightly in grains from HT-treated plants of Ardito, Grécia, and Ruivo.

**TABLE 1:** Comparison between peaks’ high of average spectra of grains from control and treated landraces plants after min–max normalization.

*Landrace*	Condition	Wavelength (cm^–1^)										
		
		Starch/proteins	Starch/fat	Fat	Proteins		Starch					
				
		OH, NH ∼3,294	CH ∼2,927	C = O ∼1,745	Amide I ∼1,648	Amide II ∼1,532	C-O_C ∼1,149	1,077	1,015	997	929	848
***Ardito***	**C**	**−**	**−**	**−**	**–**	**–**	**–**	**–**	**−**	=1	**−**	**−**
	**HT**	**+**	**+**	**+**	**++**	**++**	**++**	**++**	**+**	=1	**+**	**+**
***Grécia***	**C**	**+**	**−**	**−**	**–**	**–**	**−**	**–**	**−**	=1	**–**	**−**
	**HT**	**−**	**+**	**+**	**++**	**++**	**+**	**++**	**+**	=1	**++**	**+**
***Magueija***	**C**	**+**	**−**	**−**	**−**	**−**	**−**	**−**	**−**	=1	**−**	**−**
	**HT**	**−**	**+**	**+**	**+**	**+**	**+**	**+**	**+**	=1	**+**	**+**
***Ruivo***	**C**	**−**	**−**	**−**	**–**	**−**	**−**	**−**	**−**	=1	**−**	**−**
	**HT**	**+**	**+**	**+**	**++**	**+**	**+**	**+**	**+**	=1	**+**	**+**

*C, control plants, HT, high-temperature-treated plants. (+) and (-), higher and lower intensity, respectively, (=1) maximum at which the spectra were normalized.*

Intensity differences between spectra obtained from grains of HT-treated plants are presented in Figure 3B and showed that Ardito spectra are the most intense in all wavenumber range, except for the 929-cm^–1^ band. In the wavenumber region most related to fat with a peak at 1,745 cm^–1^ as well as two other regions more related to starch with peaks at 2,927 and 997 cm^–1^, the intensities are similar for all landraces. As for control conditions, Magueija is the landrace with lower intensity in eight of the selected spectra bands. Compared with the control, it is possible to observe more differences between the spectra of grains obtained from HT-treated plants, indicating more dissimilarities between landraces under this abiotic stress condition.

### Grain Protein Content Increase Is a Common Response to HT Treatment

**FIGURE 4:**
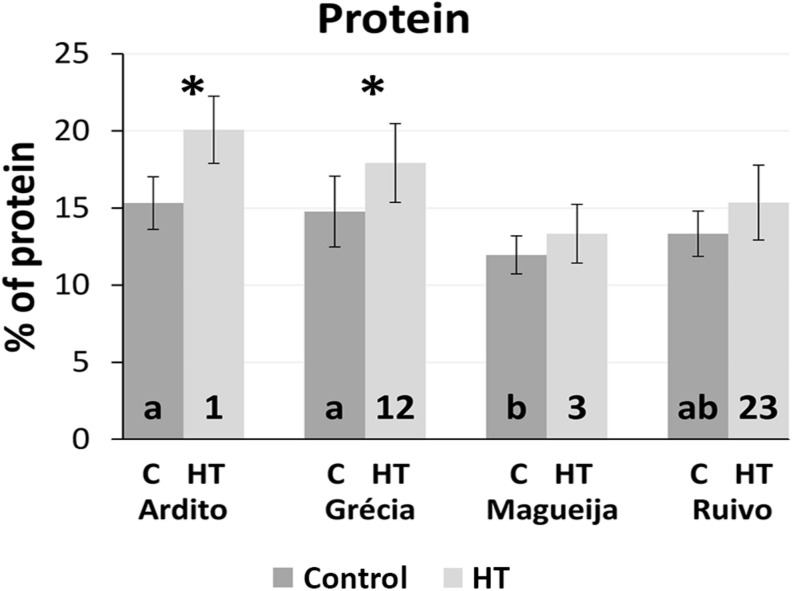
Mean protein content of plants kept in control conditions (dark gray) and HT-treated (light gray) and respective standard deviations (represented as bars). Different letters and numbers inside columns indicate *ANOVA* significant differences between varieties in control and treatment conditions (HT), respectively. *t-test statistical differences between control and treatment in each variety (*p* < 0.05).

Protein content was predicted using spectra acquired from grain of control and HT-treated plants of the four landraces using the model calibrated in Tomás et al. (2020a) adjusted with N content values of landrace grains (Supplementary Table 2). The adjusted model to predict nitrogen content had very good statistics (*R*^2^ = 0.92, RMSECV = 0.14), and the predicted nitrogen values for all control and HT samples ranged from 1.8 to 4.5% (mg of N/100 mg of flour). These values were used to infer protein content using a conversion factor of 5.7× (Caporaso et al., 2018). The average protein content of grains from control and HT-treated plants of the four landraces studied are summarized in Figure 4.

Considering the values obtained from plants kept in control conditions for each landrace, Magueija samples are the ones with the lower mean protein content (12%), significantly different from Ardito and Grécia with 15.3 and 14.8%, respectively (Figure 4). Grains of HT-treated plants showed higher protein content in all landraces analyzed in comparison with control being this augment significant in Ardito (20.1%) and Grécia (17.9%). The comparison between landraces of mean protein content obtained in HT-treated plants showed a higher value in Ardito, which is significantly different from those of Ruivo (15.4%) and Magueija (13.3%) (Figure 4).

**FIGURE 5:**
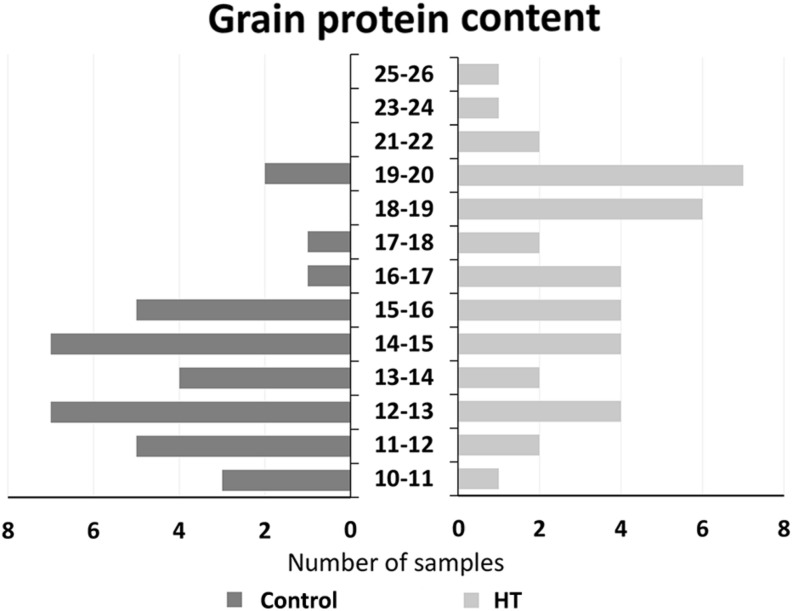
Grain protein content. Distribution of all grain samples protein content from control (dark gray) and HT-treated (light gray) plants of the four landraces studied.

A global perspective of protein content in all analyzed samples is presented in Figure 5 that represents the division by classes of protein content of all individual samples from control or HT-treated plants analyzed (dark and light bars, respectively), independently of the genotype. It shows that control grains presented a lower number of classes (nine classes with values ranging between 10.3 and 19.7%) than HT-treated samples (13 classes, with values ranging between 10.2 and 25.4%). Likewise, this result representation substantiates the lower average protein content of the control samples (13.8%) in comparison to the average value of the treatment samples (16.8%).

**FIGURE 6:**
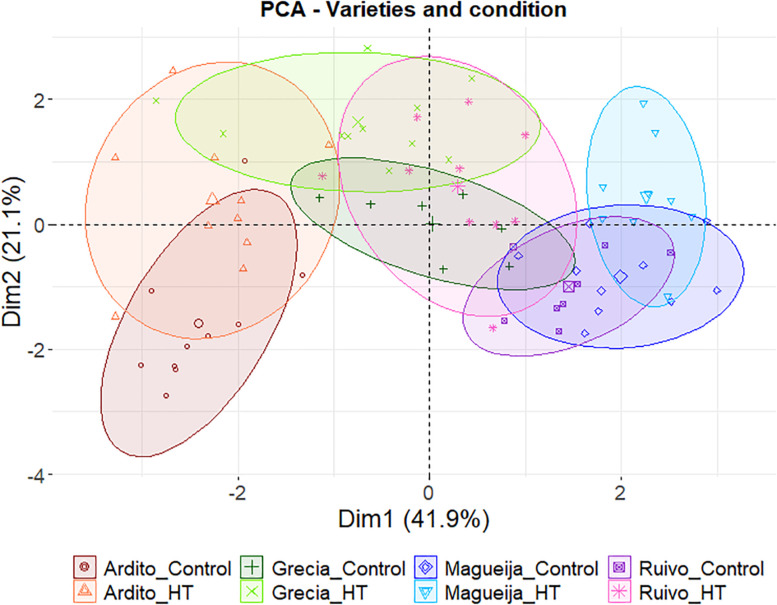
Principal component analysis using yield parameters (plant height and area, number of spikes, first spike length, and grains number and weight) and grain protein content of Ardito (red), Grécia (green), Magueija (blue), and Ruivo (pink) plants kept in control conditions (darker colors) or HT treated (lighter colors).

On the other hand, an integrated assessment of the four landraces studied can be performed through the PCA of all yield parameters and protein quantification presented in Figure 6. In this PCA, the two represented dimensions explain 63% of the variation found between samples. The first, that clearly separates Ardito and Magueija, is defined by five of the eight parameters used (plant area, spike length, grain number, grain weight, and protein content). On its turn, Ardito responds to HT privileging plant growth, increasing plant height, but reducing grain yield, with spike length, and both grain number and weight reduced in treated plants. Concerning grain composition, a significant increase in protein content was observed and, allied to the reduction in grain size and weight, foresee a reduction in grain starch amount. Also, in Magueija, the responses to HT increase plant biomass, spikes in both number and length, and grain quantity.

## Discussion

Landrace variability may assume special relevance due to commercial varieties reduced genetic diversity, constituting valuable agrobiodiversity pools potentially more adapted to local conditions where they have been cultivated for long periods (Alipour et al., 2017). Facing a global warming scenario, these advantages are even more relevant for essential crops like bread wheat considering the projections of insufficient cereal production to meet the demand in a few decades (Ray et al., 2013; Gaupp et al., 2019). In this context, the invaluable resource encompassed in the wheat old traditional landraces collected in the 1930s of the last century by Vasconcellos (1933) in Portugal fields assume special relevance. In this work, we studied four of these bread wheat landraces evaluating their yield and grain quality modulation by a HT treatment mimicking a heatwave during grain filling. This particular extreme heat event was predicted to be intensified onward especially in Portugal (Cardoso et al., 2019). Yield parameters and grain composition were comparatively evaluated in landrace plants kept in control conditions and HT treated.

The evaluation of the four landraces showed considerable intervariety diversity since significant differences were detected in all yield parameters analyzed in control plants, especially in the number of spikes, grain number, and plant height, the latter being significantly different between all the landraces. The variability disclosed in the number of grains per spike contrasts with the complete homogeneity observed in this parameter of grain yield observed in bread wheat commercial varieties (Tomás et al., 2020a). On the other hand, the diversity disclosed in the number of spikes contrasts also with the lack of diversity reported in commercial genotypes (Khan and Naqvi, 2011). Ardito landrace stands out as the one with the lower plant height and area along with the higher grain number and weight in the first spike, characteristics close to the desired for commercial varieties (Khush, 1999). Globally, the yield parameters are similar to other European landraces previously studied (Dotlaèil et al., 2003). Unexpectedly, two landraces (Magueija and Grécia) 10 grain weight was higher (0.44 g) than the higher value reported for commercial varieties recommended to be used in Portugal (0.38 g) assessed in similar assays (Tomás et al., 2020a). Concerning the average protein content, the values obtained in the landraces studied, ranging from 10.3 to 19.7% (control condition), were similar to the ones assessed in commercial varieties through the same methodology (between 9.5 and 21.4%, Tomás et al., 2020a). It is relevant that although these landraces were not submitted to breeding programs, their values of protein content are very acceptable and similar to the ones reported for commercial varieties. Recently, the screening of Pakistani wheat landraces also found several traditional genotypes with high storage protein concentration, pointing out their potential to improve the nutritional quality of modern wheat commercial genotypes (Mughal et al., 2020).

On the other hand, different landraces studied in our work revealed distinct responses to HT traduced even in opposite effects in most yield parameters evaluated. The evaluation of the plant height of Ardito and Ruivo, and the area of Magueija revealed significant differences between the control and HT plants. As plant area is often used as plant biomass predictor (Armoniené et al., 2018), our results indicate that Magueija increase in biomass may compromise grain filling, as in this plant development phase, all plant resources should be directed to grain. Both number of spikes per plant and first spike length showed a significant difference between the control and HT plants in Ardito and Magueija, although HT induced differences in the first spike length in opposite ways in these two landraces. Additionally, the increase in Magueija number of spikes induced by HT treatment was mainly due to the appearance of new tillers with spike during ripening. The appearance of these late tillers was observed in all the landraces, in both control and HT-treated plants. It must be emphasized that late spikes were never observed in commercial varieties previously assayed in similar conditions (Tomás et al., 2020a). Moreover, extemporaneous tiller appearance was described in some wheat varieties but only until the beginning of stem elongation (Bowden et al., 2007). We speculate that this phenomenon can constitute a strategy to assure descendance in extreme conditions.

The less affected yield parameter was grain number since it was the only one that did not reveal significant differences between the control and HT-treated plants in any of the genotypes assessed, in opposition to 10-grain weight, which was significantly lower in HT-treated plants of all four landraces. This is in accordance with some previous works that reported that grain number is mostly affected by HT treatments imposed before fertilization, while elevated temperature occurring during grain filling is known to shorten developing period and lead to shrunken grains (Stone and Nicolas, 1995; Farooq et al., 2011; Talukder et al., 2014; Tao et al., 2018). Although an increase in assimilate supply was reported in this phase, it was not sufficient to fully compensate the shorter duration of grain filling period (Lobell et al., 2012). Contrary to this uniform effect on grain weight observed in all landraces analyzed, some previous works revealed different HT effects in grain weight between distinct genotypes (Scotti-Campos et al., 2011; Tomás et al., 2020a). Globally, the comparative evaluation of yield-related traits between genotypes in the control and HT treatment plants showed that parameters determinant for grain yield like spike number and 10-grain weight presented high variability in both developmental conditions assayed.

Concerning grain composition evaluated by ATR-FTIR, all spectra here obtained were similar to the ones already described in Tomás et al. (2020a) for commercial wheat varieties and were concordant with the main components of wheat grain—starch and proteins (Shewry, 2009). The balance between starch and the other components suggests that grains from Ardito plants have higher protein content than the other four landraces, especially due to the contribution of Amide I (1,648 cm^–1^). Lipid fraction constitutes only 3–4% of the whole grain (Wrigley et al., 2009), and in our work, it is negligible as the embryo, which is responsible for one-third of the wheat grain lipid fraction that was removed before grain milling. Nevertheless, the comparison between average spectra shows that Magueija grains have the higher fat amount.

Overall, spectra from HT-treated samples were more intense than the control ones in all the landraces, indicating an increase in protein content and a decrease in starch. These results are in accordance with model predicted protein content, which shows a significant increase in Ardito and Grécia grains from HT-treated plants and with previous works (DuPont et al., 2006; Zhang et al., 2017; Tao et al., 2018). Also, an increase in protein content should be related with a decrease in starch content and this is in accordance with the decrease in 10-grain weight and grain size previously observed and with other studies showing that HTs affect the starch synthesis in wheat grain (Hurkman et al., 2003; Tomás et al., 2020b). A shift between landrace spectra in bands mainly assigned to starch suggests that also the proportions of different polysaccharides are altered as the effect of HT treatment. This effect was also observed in commercial genotypes submitted to similar HT treatments (Tomás et al., 2020a).

After HT treatment, the significant increase in Ardito protein amount was also reflected in the greater distance between this landrace and the other ones regarding maximum intensity at Amide I and II bands. Also, the range of maxima intensity values in each spectra band is bigger indicating differences between landraces in HT-treated plants not observed in control ones. This is also corroborated by the increased dispersion of HT sample protein values as shown in Figure 5. In fact, associated with the global increase in protein content induced by HT treatment, a higher range of protein content values was obtained after HT (15%) in comparison to control samples (9%). Even more important was the increase in protein observed in HT-treated plants that corroborate the relevance of identifying variable wheat genotypes more adapted to global warming, particularly concerning the major determinant of grain quality—protein content (Asseng et al., 2019). Additionally, the comparison of protein content range in grains from plants submitted to heatwave like the treatment here observed in landraces (10.2–25.4%) and reported in commercial genotypes (10.1–17.6%, Tomás et al., 2020a). This diversity, together with the higher average protein content observed in landraces after HT treatment, supports the relevance of old traditional genotypes as a source of useful variability breeding focused in wheat nutrimental quality.

Altogether, the four landraces studied presented clear distinct pathways in HT response testifying once again the diversity enclosed in the old varieties studied. Grécia and Ruivo are both affected in vegetative growth and yield, with a reduction, although not always significant, in almost all the parameters. The other two landraces—Magueija and Ardito—showed opposite behaviors, as unraveled by the PCA of all yield parameters and protein quantification (Figure 6). Magueija plants seem to be less affected by heatwave-like treatment in terms of yield as after HT, 10-grain weight is higher even when compared with commercial varieties. However, no significant increase in grain protein content was induced by HT, suggesting that the increase in tillers’ number may reduce the allocation of resources to the grain filling per spike, ultimately resulting in worst flour quality (Li et al., 2016; Yang et al., 2019). On the other hand, Ardito not only revealed the higher protein content in the control condition but also disclosed a significant increase in this grain quality parameter after HT treatment. Moreover, Ardito is the earlier landrace (Supplementary Table 1), with a number of days from germinations to flowering similar to the ones previously observed (not published) in commercial varieties studied in Tomás et al. (2020a), which may be determinant to avoid heat stress conditions.

The overall diverse outcomes induced by a heatwave-like treatment in distinct landraces contrasts with the reduced diversity observed in wheat commercial varieties submitted to a similar treatment previously reported (Tomás et al., 2020a,b). This superior variability, unraveled under extreme thermal conditions, highlights the potential usefulness of the biodiversity enclosed in old traditional wheat genotypes facing climate changes already sensed. Moreover, the integrative assessment of this work outcomes suggests that both Magueija and Ardito genotypes should be further evaluated seeking for attractive genotypes for wheat breeding plans.

## Data Availability Statement

The original contributions presented in the study are included in the article/Supplementary Material. Further inquiries can be directed to the corresponding author/s.

## Author Contributions

DT and MS conceptualized and validated the study. DT, JR, and MS were in charge of the methodology. DT and JR made the formal analysis. DT and LC took part in the investigation. DT wrote and prepared the original draft and was in charge of the visualization. JR, WV, and MS wrote, reviewed, and edited the manuscript. MS supervised the study, was in charge of the project administration, and acquired the funding for the study. All authors contributed to the article and approved the submitted version.

## Conflict of Interest

The authors declare that the research was conducted in the absence of any commercial or financial relationships that could be construed as a potential conflict of interest.
