# Supplementary material for: Assessment of Four Portuguese Wheat Landrace Diversity to Cope With Global Warming
Source: Front Plant Sci. 2020 Dec 9;11:594977. doi: 10.3389/fpls.2020.594977 (PMC7756116; doi:10.3389/fpls.2020.594977)
Supplement: Supplementary file 1 [file Data_Sheet_1.pdf]

Supplementary Material

Supplementary Table 1. Flowering time (days from germinations to flowering) of wheat landraces studied.

Wheat landraces	**Days until flowering**
Ardito	126±3
Grécia	140±6
Magueija	157±3
Ruivo	129±4

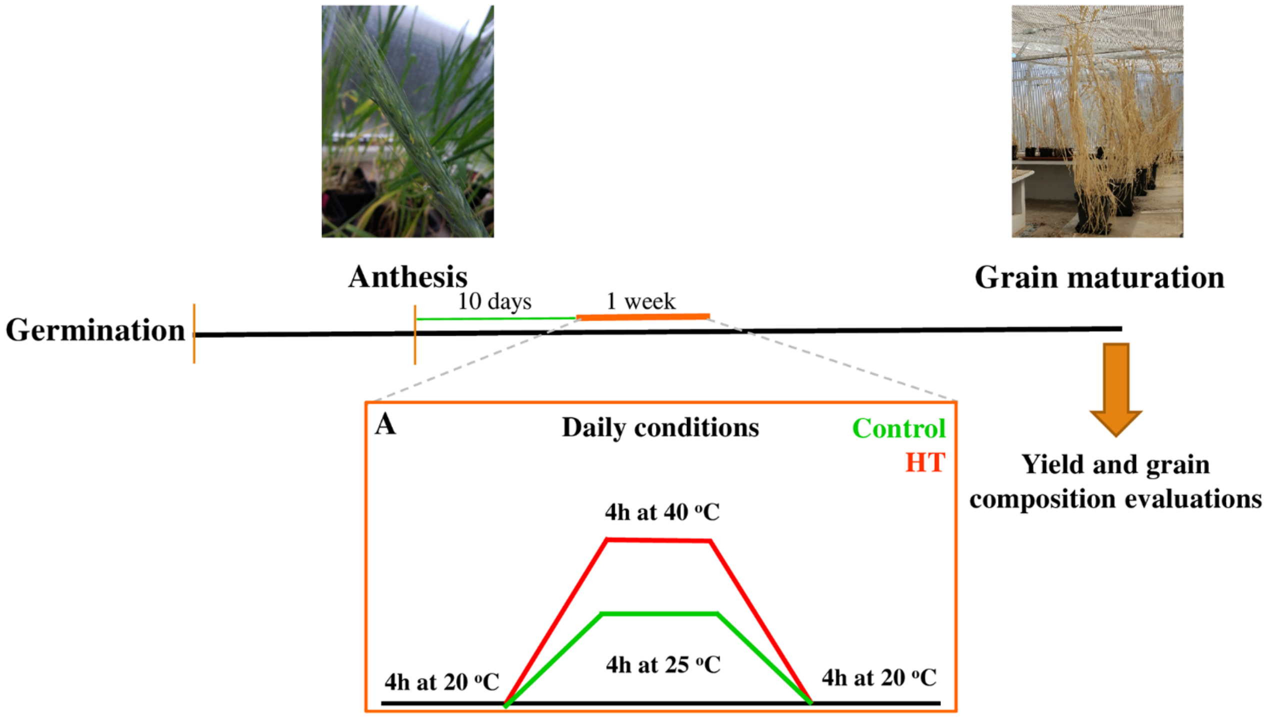

Supplementary Figure 1. High temperature assay schematic representations.

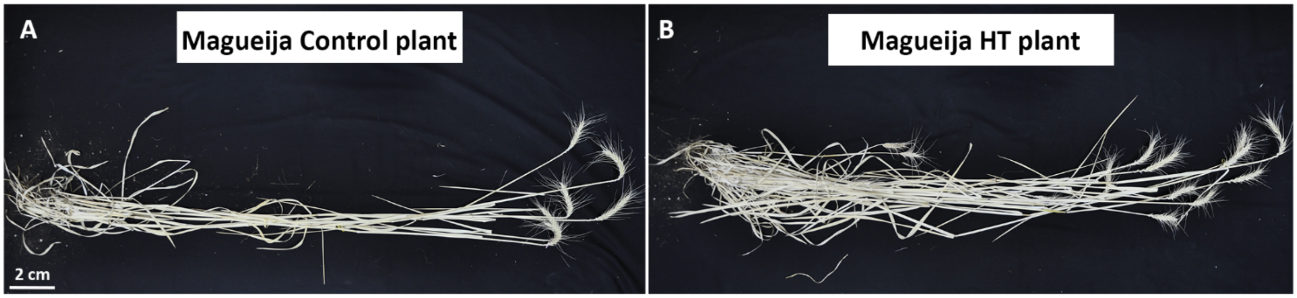

Supplementary Figure 2. Wheat Magueija mature plants **(A)** kept in control conditions and **(B)** high temperature (HT) treated.

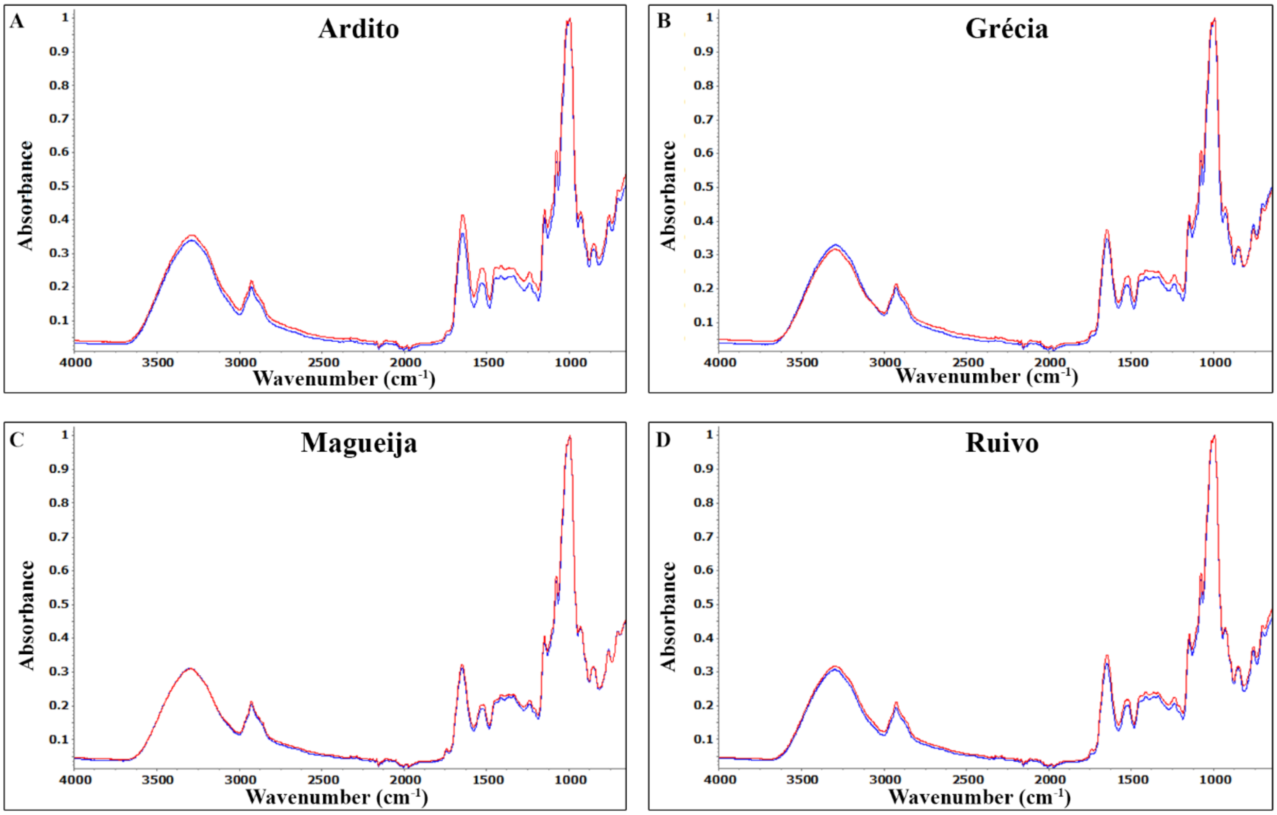

Supplementary Figure 3. Average ATR-FTIR spectra of flour from control (blue) and HT treated (red) plants of wheat landraces **(A)** Ardito, **(B)** Grécia, **(C)** Magueija and **(D)** Ruivo.

Supplementary Table 2. Nitrogen content of flour samples from plants kept in control conditions and high temperature (HT) treated, quantified by Elemental Analysis.

**Sample**	**Nitrogen content (mg of N/ 100 mg of flour)**	
	Predicted by model	Quantification by elemental analysis
Ardito HT	4.7	4.5
Ardito HT	4.2	3.8
Grécia HT	3.9	3.4
Ardito HT	3.7	2.9
Ardito Control	3.5	2.8
Ardito Control	3.2	2.6
Grécia HT	3.1	2.7
Magueija HT	2.8	2.5
Ruivo HT	2.4	2.1
Magueija HT	2.0	2.1
